# Application of Amorphous Nanomaterials in Dentistry: A Comprehensive Review

**DOI:** 10.3390/jfb17010011

**Published:** 2025-12-23

**Authors:** Iris Xiaoxue Yin, John Yun Niu, Veena Wenqing Xu, Ollie Yiru Yu, Irene Shuping Zhao, Chun Hung Chu

**Affiliations:** 1 Faculty of Dentistry, The University of Hong Kong, Hong Kong SAR, China; irisxyin@hku.hk (I.X.Y.); niuyun@hku.hk (J.Y.N.); veenaxu@hku.hk (V.W.X.); ollieyu@hku.hk (O.Y.Y.); 2 School of Dentistry, Shenzhen University Medical School, Shenzhen 518055, China

**Keywords:** amorphous, nanomaterials, dentistry, non-crystalline, calcium

## Abstract

Conventional dental materials with organised crystal structures exhibit limitations in corrosion resistance, bioactivity, and drug delivery capability. In contrast, amorphous nanomaterials offer potential advantages in overcoming these limitations due to their unique structural properties. They are characterised by a non-crystalline, disordered atomic structure and are similar to a solidified liquid at the nanoscale. Among the amorphous nanomaterials used in dentistry, there are five major categories: calcium-, silicon-, magnesium-, zirconia-, and polymer-based systems. This study reviewed these amorphous nanomaterials by investigating their synthesis, properties, applications, limitations, and future directions in dentistry. These amorphous nanomaterials are synthesised primarily through low-temperature methods, including sol–gel processes, rapid precipitation, and electrochemical etching, which prevent atomic arrangements into crystalline structures. The resulting disordered atomic configuration confers exceptional properties, including enhanced solubility, superior drug-loading capacity, high surface reactivity, and controlled biodegradability. These characteristics enable diverse dental applications. Calcium-based amorphous nanomaterials, particularly amorphous calcium phosphate, demonstrate the ability to remineralise tooth enamel. Silicon-based amorphous nanomaterials function as carriers that can release antibacterial agents in response to stimuli. Magnesium-based amorphous nanomaterials are antibacterial and support natural bone regeneration. Zirconia-based amorphous nanomaterials strengthen the mechanical properties of restorative materials. Polymer-based amorphous nanomaterials enable controlled release of medications over extended periods. Despite the advances in these amorphous nanomaterials, there are limitations regarding material stability over time, precise control of degradation rates in the oral environment, and the development of reliable large-scale manufacturing processes. Researchers are creating smart materials that respond to specific oral conditions and developing hybrid systems that combine the strengths of different nanomaterials. In summary, amorphous nanomaterials hold great promise for advancing dental treatments through their unique properties and versatile applications. Clinically, these materials could improve the durability, bioactivity, and targeted drug delivery in dental restorations and therapies, leading to better patient outcomes.

## 1. Introduction

Nanotechnology has been predominantly built upon crystalline materials in the past decades, where atoms are arranged in a long-range, repeating order. Amorphous nanoparticles represent a distinct class of materials that are attracting considerable attention due to their unique structural characteristics and associated properties [1]. In contrast to crystalline materials, amorphous nanomaterials exhibit a non-crystalline, disordered atomic structure, resembling a solidified liquid at the nanoscale [2]. This fundamental lack of long-range periodic order is the origin of their exceptional and advantageous properties. The primary outcome of this disordered structure is structural isotropy, characterised by uniform properties in all directions resulting from the lack of grain boundaries and directional crystal planes. This isotropy results in improved mechanical reliability, consistent performance, and uniform dissolution behaviour [3]. It is critical for applications requiring consistent wear, such as in durable dental fillings or protective coatings. Furthermore, the disordered atomic arrangement creates a high density of unsaturated bonds and defects at the surface, resulting in exceptionally high surface energy and reactivity [4]. Thus, amorphous nanomaterials possess superior catalytic activity and enhanced bioactivity. Their highly reactive surface promotes strong adhesion to biological tissues and provides abundant active sites for beneficial interactions, such as facilitating the nucleation of hydroxyapatite for enamel remineralisation or generating antibacterial agents. Furthermore, the absence of a constraining crystal lattice grants these materials a superior capacity for tunable solubility and high drug loading [5]. Unencumbered by a slow-dissolving crystalline structure, bioactive compounds can release therapeutic ions at significantly higher concentrations and rates to combat demineralisation. The loose atomic packing facilitates the incorporation and controlled release of substantial payloads of various therapeutic agents, including antibiotics and anti-inflammatory drugs, thereby enabling sophisticated sustained-release platforms for targeted treatment [6]. The combination of isotropy, heightened reactivity, and improved drug-loading capacity, along with the ability to tailor composition without crystalline constraints, establishes amorphous nanomaterials as a versatile and innovative platform for advanced technologies, particularly in dentistry.

The quest for optimal dental materials that effectively integrate with the complex oral environment, providing superior mechanical properties, increased bioactivity, and exceptional clinical longevity, constitutes a significant challenge in the contemporary dentistry [7]. This effort is fundamentally motivated by the necessity to address widespread oral diseases such as dental caries. Dental caries represents a significant global health issue, impacting a large portion of the population [8]. For decades, crystalline-based materials have underpinned the field, including amalgam alloys, ceramic implants, and fluoride-releasing compounds, which constitute the foundation of restorative and preventive care [9]. The crystalline structure, defined by a long-range ordered atomic lattice, inherently imposes fundamental limitations [10]. These materials demonstrate a natural vulnerability to acid corrosion in the variable pH environment of the oral cavity, resulting in the deterioration of restorations and the development of secondary caries [11]. The presence of grain boundaries and structural defects within matrices serves as microscopic stress concentrators, leading to crack initiation and compromising mechanical integrity and fatigue resistance [12]. The release of therapeutic ions for remineralisation from various crystalline systems is frequently self-limiting and inadequate to surpass the demineralisation process caused by cariogenic biofilms. Additionally, their rigid lattice structure inherently limits effective drug-loading capacity [13]. The interplay of corrosion, mechanical failure, and limited bioactivity undermines the long-term efficacy of dental treatments, frequently resulting in an expensive cycle of repeated restorations [14,15,16]. The ongoing disparity between the theoretical capabilities of conventional materials and their practical limitations highlights the critical necessity for a transformative paradigm.

**Figure 1:**
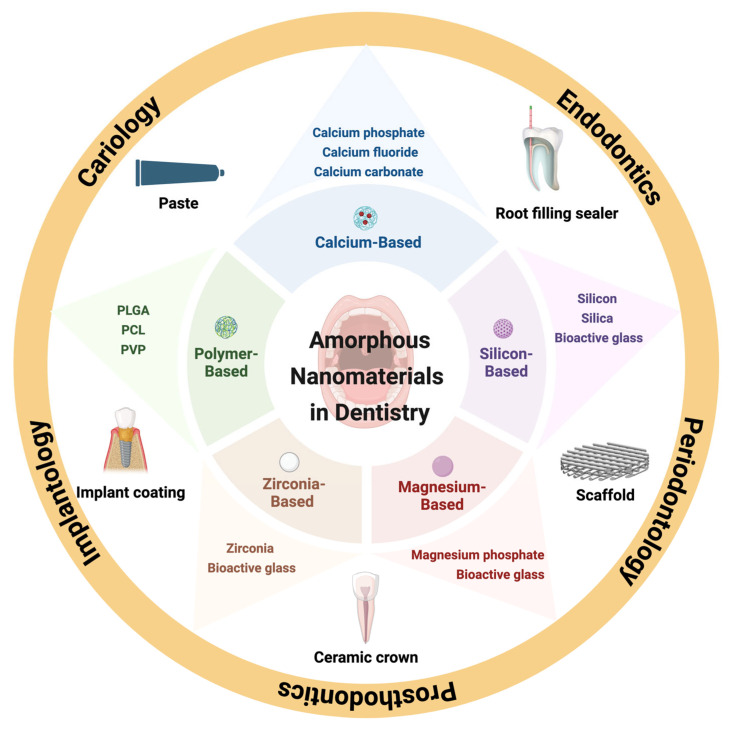
Applications of amorphous nanomaterials in dentistry.

Amorphous nanomaterials have emerged as a significant alternative in response to these challenges, with the potential to transform dental therapeutics (Figure 1). The disordered atomic structure provides specific advantages that mitigate the limitations and advance the field of preventive and restorative dentistry [17,18]. Structural isotropy guarantees uniform strength and dissolution behaviour [19]. Elevated surface energy and reactivity improve bio-adhesion and bioactivity. Importantly, the lack of a confining crystal lattice enables a greater loading of therapeutic agents (e.g., antimicrobials, remineralising ions) and supports a more controllable and sustained release profile [16,20]. The utilisation of these materials in dentistry is expanding swiftly. Amorphous calcium phosphate nanoparticles are not limited by the rate of a crystalline lattice [21]. They can release calcium and phosphate ions more efficiently than crystalline phases, facilitating remineralisation to counteract caries [22]. Amorphous silica nanoparticles serve as effective carriers for antibacterial agents such as silver or chlorhexidine, offering prolonged protection within biofilms [23,24]. Amorphous nanomaterials are being integrated into composites and adhesives within restorative dentistry to enhance mechanical strength, minimise polymerisation shrinkage, and improve bonding integrity [25,26].

Given the significant potential of amorphous nanomaterials, there are increasing number of studies exploring their applications in dentistry. This expanding field currently lacks a synthesised and critical overview that consolidates these findings into a coherent framework. Therefore, the objective of this study is to comprehensively review the application of amorphous nanomaterials in dentistry.

## 2. Calcium-Based Amorphous Nanomaterials

The battle against dental caries, a demineralisation process of the dental hard tissues initiated by acid-producing biofilms, has found a potent ally in calcium-based amorphous nanomaterials [27]. Among these, amorphous calcium phosphate stands as the most extensively researched and clinically translatable representative [28]. Its paramount significance in dentistry stems from its role as a highly bioactive precursor phase to hydroxyapatite, the primary mineral component of teeth and bones [29]. Unlike its crystalline counterparts (e.g., hydroxyapatite, fluorapatite), amorphous calcium phosphate lacks a long-range atomic order, which confers a critical thermodynamic instability [30]. This metastability is not a weakness but the very source of its exceptional bioactivity. Amorphous calcium phosphate drives a rapid, spontaneous transformation toward a more stable crystalline phase in aqueous environments, such as the oral cavity or within a moist dental composite [31]. While amorphous calcium phosphate dominates the landscape, other amorphous calcium-based materials are emerging. Amorphous calcium carbonate is being explored for its high solubility and potential as an alternative calcium source [32,33], while amorphous calcium fluoride phosphate is particularly exciting as its co-releases fluoride and phosphate ions, offering a synergistic remineralisation and antibacterial effect [34,35,36].

**Figure 2:**
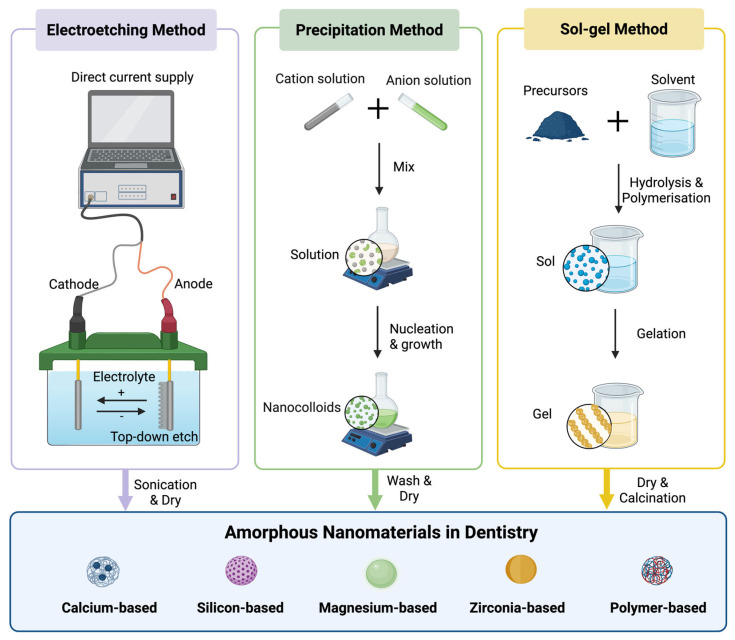
Synthesis of amorphous nanomaterials in dentistry.

The synthesis of amorphous calcium-based nanoparticles is typically achieved through rapid precipitation from supersaturated solutions of calcium and phosphate ions (Figure 2). This process kinetically traps the atoms in a disordered state and prevents the thermodynamically favoured crystallisation [37]. Common methods include straightforward wet chemical precipitation, often under tightly controlled alkaline conditions, and more advanced sol–gel techniques that offer superior particle size control [38]. The critical parameters of pH, temperature, and ionic concentration must be meticulously managed, as slight deviations can lead to the instantaneous transformation into undesirable crystalline phases like octacalcium phosphate or hydroxyapatite [37].

A key challenge of amorphous calcium-based materials’ synthesis and clinical use is their inherent tendency to crystallise [39]. Therefore, stabilisation strategies are paramount. Common approaches include the incorporation of biocompatible stabilisers and integration into matrices. The incorporation of biocompatible stabilisers is the most common approach. Molecules such as poly (acrylic acid), citrate, or carboxylate polymers adsorb onto the surface of nascent amorphous calcium-based nanoparticle nuclei via their functional groups, creating a strong electrostatic or steric barrier that inhibits ion addition and crystal growth [40,41,42,43,44]. The most prominent commercial example is casein phosphopeptide-amorphous calcium phosphate (CPP-ACP). In this complex, the phosphopeptides derived from milk casein bind to amorphous calcium phosphate clusters, effectively sequestering them and preventing crystallisation, thus maintaining a bioavailable calcium phosphate reservoir in products like sugar-free gums and topical creams [45,46,47,48]. For restorative applications, amorphous calcium-based nanoparticles are directly incorporated into resin composites, adhesives, or varnishes [49,50,51]. Here, the polymer network itself acts as a physical barrier, slowing the diffusion of water and ions to the amorphous calcium-based nanoparticle fillers, thereby providing a controlled, long-term release rather than an immediate burst [52].

The therapeutic efficacy of calcium-based amorphous nanomaterials is directly linked to their unique dissolution-reprecipitation pathway, which enables a biomimetic repair process [53]. The primary mechanism is the creation of a highly supersaturated local environment [54]. When calcium-based amorphous nanomaterials are exposed to saliva or carious lesions, they release high concentrations of calcium (Ca^2+^) and phosphate (PO_4_^3−^) ions. This ion-rich milieu penetrates the porous body of early enamel lesions and drives the deposition of apatitic crystals, effectively reversing the demineralisation process and restoring tissue hardness [55]. This mechanism enables calcium-based amorphous nanomaterials to be more efficient than the simple deposition of pre-formed, large hydroxyapatite crystals [56].

Studies demonstrated amorphous calcium phosphate is a key enabler of biomimetic mineralisation [57]. The biological formation of dental enamel is a highly controlled process orchestrated by amelogenin proteins [58]. These proteins stabilise amorphous calcium phosphate precursors, guiding their infiltration and transformation into the intricate, aligned crystalline architecture of mature enamel [59]. Amorphous calcium phosphate nanomaterials effectively replicate this precursor-based strategy. Amorphous calcium phosphate precipitates as apatite crystals, leading to the epitaxial growth of new mineral that is seamlessly integrated with the old. Biomimetic mineralisation using amorphous calcium phosphate aims to regenerate the lost tooth structure from within, offering distinct advantages over conventional remineralisation [60]. It effectively remineralises the entire body of the lesion, not just the surface, preventing a brittle surface layer from masking a weakened subsurface [61,62]. The newly formed mineral is structurally aligned with the native tissue, leading to superior mechanical properties and aesthetics compared to randomly deposited crystals [62].

Calcium-based amorphous nanomaterials have been successfully incorporated into a wide range of dental materials, including restorative composites, bioactive adhesives, and prophylactic pastes and [27,40,63,64]. Amorphous calcium phosphate-filled composites represent a shift from passive to active restorations. They act as rechargeable ion reservoirs, not only releasing remineralising ions but also being “recharged” by exposure to fluoride from toothpaste [65]. A significant research focus is optimising the amorphous calcium phosphate filler load to maximise bioactivity without compromising the composite’s mechanical strength and wear resistance, often by using core–shell structures or hybrid fillers [66,67,68]. Incorporating amorphous calcium phosphate into dentin adhesives is a strategic approach to combating the Achilles’ heel of restorations: the hybrid layer [69,70]. By creating a bioactive interface, the adhesive can help remineralise the exposed dentin collagen and seal the marginal gaps, significantly reducing the risk of secondary caries and prolonging the restoration’s lifespan. Amorphous calcium phosphate-based varnishes are used to treat hypersensitivity and early caries by occluding dentinal tubules and depositing a protective mineral layer on the tooth surface [71]. Beyond restoratives, the application of amorphous calcium phosphate extends to orthodontic care, where varnishes or adhesives can prevent white spot lesions around brackets [56]. Amorphous calcium phosphate is added into sealer additives to promote periapical tissue healing in endodontics [72].

Despite the promise, challenges remain. The fundamental trade-off between the bioactivity of calcium-based amorphous nanomaterials and the mechanical properties of the resulting composite remains a key hurdle. The long-term stability of calcium-based amorphous nanomaterials within polymeric matrices can be a concern, as premature crystallisation can deactivate the ion reservoir [73]. Furthermore, optimising the ion release profile to match the dynamic challenges of the oral biofilm is an area of active research. Future directions include the development of multi-functional amorphous calcium-based nanoparticles co-loaded with fluoride or antimicrobial agents (e.g., silver, zinc) to simultaneously provide remineralisation and antibacterial action [28,74]. The exploration of other amorphous calcium compounds, such as amorphous calcium carbonate or amorphous calcium fluoride phosphate, also presents an exciting frontier for creating next-generation, multifunctional bioactive dental materials.

## 3. Silicon-Based Amorphous Nanomaterials

Silicon-based amorphous nanomaterials represent a pivotal and highly versatile class of materials in advanced dentistry [75]. This category primarily encompasses two distinct yet related subgroups: amorphous silicon, which is the elemental form, and amorphous silica, which are oxides of silicon. Their properties and primary dental functions diverge significantly, offering a complementary toolkit for therapeutic delivery, reinforcement, and tissue regeneration.

### 3.1. Amorphous Silicon Nanomaterials

The most clinically relevant form of amorphous silicon nanomaterials is porous silicon, typically produced by the electrochemical etching of crystalline silicon wafers (Figure 2). This process allows for unparalleled control over the material’s nanostructure [76]. The pore size, volume, and layer structure of amorphous silicon nanoparticles can be precisely engineered, creating a high-surface-area sponge-like matrix [77]. This structure is ideal for loading high payloads of therapeutic agents. Furthermore, the surface of freshly etched amorphous silicon nanoparticles is hydride-terminated (Si-H), which can be readily functionalised with various organic molecules to control its stability, reactivity, and biological interactions [77]. Amorphous silicon nanoparticles can be degraded into orthosilicic acid [Si(OH)_4_] in physiological environments, which is the natural, bioavailable form of silicon found in the body [78]. This resorbability is a critical advantage over permanent silica or polymer carriers, as it eliminates the long-term fate concerns of the nanomaterial itself [79]. The degradation rate can be precisely tuned from days to months by controlling the porosity, surface chemistry, and degree of thermal oxidation [80].

The primary application of amorphous silicon nanomaterials in dentistry is as a smart, resorbable drug delivery vehicle [81]. For periodontal therapy, amorphous silicon nanomaterials can be loaded with anti-inflammatory agents or antibiotics [82]. When placed in a periodontal pocket, they provide sustained release of the drug over a period of weeks as the silicon matrix gradually dissolves, supporting tissue healing and then safely disappearing [83]. For vital pulp therapy, amorphous silicon nanomaterials can release odontogenic growth factors or antimicrobials directly at the pulp-dentin interface. Its biodegradability ensures no permanent barrier remains, potentially facilitating better natural tissue repair [84]. In root canals, amorphous silicon nanoparticles incorporated into a sealer could provide prolonged intracanal disinfection [85,86]. The osteogenic potential of silicon makes amorphous silicon nanomaterials the promising biomaterial for oral and maxillofacial surgery as bioactive scaffolds and resorbable implant coatings [87]. As amorphous silicon nanomaterials degrade, they release orthosilicic acid, which has been shown to stimulate osteoblast proliferation and collagen type I formation [87,88]. This actively promotes bone regeneration around dental implants or in mandibular defects [89].

The translation of amorphous silicon nanomaterials faces specific hurdles. The high cost and the challenge of scaling up the manufacturing of sterilised, clinical-grade amorphous silicon nanomaterials are key hurdles [90]. The long-term biological response to high local concentrations of degradation products, while generally considered safe, requires further detailed study. Future research is directed towards developing hybrid materials to achieve more sophisticated release profiles.

### 3.2. Amorphous Silica Nanomaterials

Amorphous silica nanomaterials are typically biocompatible and offer a synthetic flexibility that can be precisely engineered for specific dental applications [91]. The most common synthesis methods of amorphous silica nanoparticles include the Stöber process, which produces monodisperse, solid silica nanoparticles, and sol–gel techniques, which can be adapted to create mesoporous silica nanoparticles with ordered pore networks (Figure 2) [92,93,94,95]. Mesoporous silica nanoparticles often present a honeycomb-like structure with pore diameters typically ranging from 2 to 10 nm [96]. This high surface area provides a vast reservoir for loading therapeutic agents in dentistry. Furthermore, the abundant silanol (Si-OH) groups on the amorphous silica nanoparticle surface serve as anchors for a wide range of functional molecules [97,98]. The surface can be grafted with polymers that respond to specific stimuli in the oral environment [99].

The application of mesoporous amorphous silica nanomaterials as controlled-release systems is their most transformative contribution to dentistry [28]. Amorphous silica nanomaterials can be loaded with high doses of chlorhexidine or silver ions for caries management [28,67]. When functionalised with pH-responsive linkers, these nanoparticles remain stable at neutral pH but rapidly release their antibacterial cargo in the acidic microenvironment of a dental plaque biofilm, enabling targeted eradication of cariogenic pathogens without indiscriminately affecting the oral microbiome [24,28,99,100]. For periodontitis, amorphous silica nanomaterials can be incorporated into gels or fibres placed in periodontal pockets, providing sustained release of antibiotics or anti-inflammatory agents like host-modulatory drugs [100,101,102]. In root canal therapy, MSNs doped with antibiotics and mixed into sealers can help disinfect the complex dentinal tubule network, significantly reducing the risk of reinfection [85,103].

Beyond delivery, amorphous silica nanoparticles are used as fillers in dental composites and adhesives [104,105,106,107]. Due to their nanoscale size and high surface area, silica nanoparticles can effectively infiltrate the resin matrix, forming a dense, interpenetrating network [108]. This leads to significant improvements in mechanical properties, including tensile strength, modulus of elasticity, and resistance to wear, thereby extending the service life of the restoration [28,75,106,107,109]. The incorporation of nanofillers reduces the overall volume of the polymerisable resin matrix, directly mitigating the detrimental polymerisation shrinkage stress that can lead to marginal gaps, microleakage, and secondary caries [67,104,108]. Silane coupling agents, which are molecular bridges containing silica-like components, are essential for bonding resin-based composites to the silica-containing ceramic fillers and the collagen network of dentin [75,110,111]. Furthermore, primer solutions containing silica nanoparticles can infiltrate and reinforce the demineralised dentin collagen layer, creating a stronger and more durable bond. Amorphous silica nanomaterials are instrumental in surface engineering of dental implants and prostheses [93,95,112]. Sol–gel-derived silica coatings can be applied to titanium implants. These coatings can be doped with bioactive ions (e.g., strontium for osteogenesis and silver for antimicrobial capacity) to enhance osseointegration and prevent peri-implantitis [24].

Despite their promise, the translation of amorphous silica nanomaterials faces several hurdles. The long-term biological fate of high doses of nanoparticles, especially their biodistribution and potential for inflammatory responses, requires thorough investigation [113]. The cost-effective, large-scale production of uniformly functionalised amorphous silica nanomaterials for clinical use remains a challenge. Furthermore, the potential for nanoparticle agglomeration within composite resins can negate their reinforcing benefits if not properly addressed [114]. Future research is sharply focused on developing next-generation “intelligent” amorphous silica nanomaterials. This includes designing multi-functional nanoparticles that can simultaneously release antibacterial agents and remineralising ions, creating more sophisticated stimuli-responsive systems.

## 4. Magnesium-Based Amorphous Nanomaterials

Magnesium-based amorphous nanomaterials offer a unique combination of properties, positioning them as highly biocompatible and bioactive materials for regenerative dentistry [88,115,116]. The most common method to synthesise amorphous magnesium-based nanoparticles is wet chemical precipitation (Figure 2). This involves the rapid mixing of aqueous solutions of magnesium ions and phosphate ions under alkaline conditions [117]. The synthesis of magnesium-based amorphous nanomaterials, particularly amorphous magnesium phosphate, requires precise control over reaction kinetics to bypass the formation of stable crystalline phases like struvite or newberyite [25,118,119].

Magnesium-based amorphous nanomaterials offer a unique combination of biodegradability, osteogenic potential, and antimicrobial properties [120]. Magnesium-based amorphous nanomaterials, such as amorphous magnesium phosphate, degrade in the physiological environment, releasing Mg^2+^ ions [120,121]. This controlled dissolution is a key advantage for temporary scaffolds or coatings that are designed to be resorbed. The released Mg^2+^ ions can stimulate osteoblast proliferation and activity [122]. This promotes enhanced bone regeneration and osseointegration, which is critical for the success of dental implants and the healing of periodontal bone defects. Magnesium degradation is accompanied by a localised, transient increase in pH, creating an alkaline environment that is inhibitory to cariogenic and periodontal pathogens. This provides a non-antibiotic antimicrobial effect [119,120].

The properties of amorphous magnesium nanomaterials translate into several promising applications. Amorphous magnesium nanomaterials can be consolidated into scaffolds or used as a coating on titanium implants [25,115,116,123]. As the amorphous coating degrades, it releases Mg^2+^ ions that stimulate rapid bone growth around the implant, while the coating itself safely disappears, eliminating any long-term interface issues [124]. Injectable hydrogels or membranes loaded with amorphous magnesium nanomaterials can be applied to periodontal defects or extraction sockets [115]. The sustained release of Mg^2+^ ions would simultaneously create a less favourable environment for oral pathogens and actively encourage the regeneration of the alveolar bone. The primary challenge and future research lie in controlling the degradation rate of the highly reactive amorphous magnesium nanomaterials to match the kinetics of tissue healing, preventing too-rapid dissolution that could compromise mechanical integrity too early.

## 5. Zirconia-Based Amorphous Nanomaterials

Zirconia is widely used in dentistry because of its strong, crystalline tetragonal form [125,126,127]. Its amorphous counterpart is engineered not to replace crystalline zirconia but to complement it by enabling new functionalities. The synthesis of amorphous zirconia nanoparticles typically involves low-temperature pathways that kinetically hinder crystallisation [128]. Common methods include sol–gel synthesis, using zirconium alkoxide precursors, and precipitation methods, using zirconium salt solutions. Stabilising the amorphous phase often requires the incorporation of dopants like silicon or phosphorus, or surface capping with organic ligands that prevent atomic rearrangement [129].

The amorphous structure of zirconia nanomaterials confers advantages of microstructural control [130]. The addition of amorphous zirconia nanoparticles facilitates the creation of fully dense zirconia ceramics with finer grain sizes and fewer defects. This leads to improved mechanical reliability and optical properties for monolithic restorations like crowns and bridges [31,131]. The integration of amorphous zirconia nanoparticles into dental resin composites can significantly enhance their fracture toughness, hardness, and wear resistance, leading to more durable posterior restorations [132]. Additionally, the intrinsic bioactivity of amorphous zirconia nanoparticles contributes to the improvement of osseointegration when coated in implants or integrated into bone graft materials. The disordered surface of amorphous zirconia is highly reactive, with a greater density of hydroxyl groups [31]. This promotes the nucleation of bone-like hydroxyapatite in physiological fluids. Future work will focus on enhancing stabilisation and exploring their synergistic use with other bioactive ions.

## 6. Polymer-Based Amorphous Nanomaterials

Polymer-based amorphous nanomaterials are tunable, serving as sophisticated carriers for therapeutic agents and as scaffolds for tissue engineering. The utility of amorphous polymer nanomaterials stems from their high drug loading and stability. Amorphous polymer nanomaterials are typically synthesised via methods like nanoprecipitation and emulsion-solvent evaporation, which allow for tight control over particle size and morphology, critical for biological interactions [133]. Their disordered molecular chains allow for the molecular-level dispersion of therapeutic agents, preventing drug crystallisation and leading to higher apparent solubility and more stable formulations. By selecting biocompatible, amorphous polymers such as poly (lactic-co-glycolic acid) (PLGA) and polyvinylpyrrolidone (PVP), the degradation rate and subsequent drug release profile can be precisely engineered, from days to several months [134,135,136].

The applications of amorphous polymer nanomaterials are broad. Amorphous polymer nanomaterials are investigated for the controlled release of antibiotics in periodontal pockets and anti-inflammatory agents for pulpal inflammation [137,138]. They can be incorporated into dental adhesives or composite resins for providing long-term release of remineralising or antibacterial agents, actively preventing secondary caries [139]. Amorphous polymer nanofibers can create non-woven, porous scaffolds that mimic the native extracellular matrix [140]. These scaffolds can be seeded with stem cells or loaded with growth factors to guide the regeneration of the dentine-pulp complex or periodontal ligament.

Key challenges in the application of amorphous polymer nanomaterials involve ensuring manufacturing reproducibility, scalable Good Manufacturing Practice production, and understanding long-term biodegradation [141]. The future research lies in developing stimuli-responsive polymers activated by oral pH or enzymatic triggers, and 4D-printed scaffolds that dynamically adapt to physiological cues. Table 1 shows the synthesis methods, key properties, potential applications, and limitations of amorphous nanomaterials used in dentistry.

## 7. Safety and Regulatory Considerations for Clinical Translation

The translation of amorphous nanomaterials from promising in vitro results to clinical dental applications necessitates rigorous safety evaluation and regulatory compliance. A primary concern of amorphous nanomaterials is the nanoparticle-specific bio-interaction. Their high surface-area-to-volume ratio and reactive surfaces, while beneficial for functionality, raise questions about long-term biological fate. Potential challenges include unintended cellular internalisation, off-target biodistribution, and the inflammatory potential of persistent nanomaterials or their agglomerates [142,143,144]. Furthermore, the very properties that define their utility—such as the controlled release of ions (e.g., Ca^2+^, Mg^2+^, Si^4+^) or the degradation of carriers like porous silicon—require careful monitoring of local and systemic concentrations to ensure they remain within therapeutic, non-cytotoxic windows. A comprehensive toxicological profile, therefore, assesses not only acute cytotoxicity but also genotoxicity, immunogenicity, and long-term organ accumulation [143].

Consequently, safety-by-design should be an integral principle from the earliest phases of material development. This proactive approach aligns with the growing emphasis from regulatory bodies worldwide, such as the U.S. FDA and the European Medicines Agency, which are actively developing frameworks for nanotechnology-based medical products. As highlighted in recent analyses of nanostructured medical devices, regulatory evaluation demands a thorough characterisation of the nanomaterial’s physicochemical properties (size, charge, porosity, stability), its biological performance, and a clear justification of its benefit-risk ratio for the intended dental application [145]. Ultimately, a clear pathway for scalable, reproducible manufacturing under Good Manufacturing Practice (GMP) is inseparable from the safety discussion, as it ensures batch-to-batch consistency—a fundamental prerequisite for predictable biological behaviour and regulatory approval.

## 8. Conclusions

The disordered atomic structure of amorphous nanomaterials, encompassing calcium-, silicon-, magnesium-, zirconia-, and polymer-based systems, confers unique properties that directly address long-standing challenges in dentistry. These materials enable numerous advanced functions, including enhanced bioactivity for biomimetic remineralisation, superior drug-loading capacity for targeted therapy, precisely controlled biodegradability for guided tissue regeneration and improved mechanical performance for durable restorations. However, the translation of these promising materials from laboratory innovation to routine clinical practice faces significant hurdles. Key challenges include ensuring the long-term stability of their metastable nature, achieving precise control over degradation kinetics, and establishing scalable, reproducible synthesis under standardised manufacturing protocols. Furthermore, a comprehensive understanding of their biological interactions and long-term fate within the dynamic oral environment, supported by systematic toxicological studies, is paramount for ensuring clinical safety and efficacy.

## Figures and Tables

**Table 1:** Synthesis, properties, applications, and limitations of key amorphous nanomaterials for dental use.

Amorphous Nanomaterials	Synthesis Methods	Key Properties	Applications in Dentistry	Limitations
**1. Calcium-Based Amorphous Nanomaterials**				
Calcium phosphate nanoparticles	Precipitation	High ion release	Composite / adhesive filler for caries prevention	Premature crystallisation
Calcium phosphate fluoride nanoparticles	Sol–gel	Enhanced mineralisation	Paste and varnish ingredient for caries prevention	Uncontrolled ion release
Calcium fluoride nanoparticles			Root filling sealer for periapical tissue healing	
Calcium carbonate nanoparticles			Desensitising agent for erosion treatment	
**2. Silicon-Based Amorphous Nanomaterials**				
Silicon nanoparticles	Sol–gel	High drug loading	Targeted drug delivery for caries prevention	Unclear long-term safety
Silica nanoparticles	Electroetching	Mechanical reinforcement	Targeted drug delivery for periodontal treatment	Potential inflammation
Bioactive glasses	Melt quenching		Composite filler for caries prevention	
			Implant coating for implantitis prevention	
			Implant coating for bone regeneration	
** 3. Magnesium-Based ** **Amorphous Nanomaterials**				
Magnesium phosphate nanoparticles	Precipitation	Controlled bio-degradability	Scaffold for bone regeneration	Uncontrolled degradation
Bioactive glasses		Osteogenic potential	Implant coating for bone regeneration	Compromise mechanical integrity
		Antimicrobial activity	Periodontal membrane for bone regeneration	
			Composite filler for caries prevention	
**4. Zirconia-Based Amorphous Nanomaterials**				
Zirconia nanoparticles	Precipitation	Mechanical reinforcement	Ceramic filler for durable restoration	Thermodynamical instability
Bioactive glasses	Sol–gel	Osteogenic potential	Composite fillers for caries prevention	
			Implant coating for bone regeneration	
			Scaffold for bone regeneration	
**5. Polymer-Based Amorphous Nanomaterials**				
Poly (lactic-co-glycolic acid) nanoparticles	Precipitation	High drug loading	Targeted drug delivery for periodontal treatment	Complex and costly production
Poly(ε-caprolactone) nanoparticles	Emulsion	Tunable degradation	Scaffold for pulp tissue engineering	Variable reproducibility
Polyvinylpyrrolidone nanoparticles		Superior Stability	Scaffold for bone regeneration	Unknown degradation products
			Composite/adhesive filler for caries prevention	

## Data Availability

No new data were created or analyzed in this study. Data sharing is not applicable to this article.
